# Anthropogenic warming is a key climate indicator of rising urban fire activity in China

**DOI:** 10.1093/nsr/nwae163

**Published:** 2024-05-07

**Authors:** Qichao Yao, Dabang Jiang, Ben Zheng, Xiaochun Wang, Xiaolin Zhu, Keyan Fang, Lamei Shi, Zhou Wang, Yongli Wang, Linhao Zhong, Yanyan Pei, Amy Hudson, Shuai Xu, Maowei Bai, Xinyan Huang, Valerie Trouet

**Affiliations:** National Institute of Natural Hazards, Ministry of Emergency Management of China, Beijng 100085, China; Key Laboratory of Sustainable Forest Ecosystem Management-Ministry of Education, School of Forestry, Northeast Forestry University, Harbin 150040, China; Laboratory of Tree-Ring Research, University of Arizona, Tucson 85721, USA; Institute of Atmospheric Physics, Chinese Academy of Sciences, Beijing 100029, China; Department of Statistics, Colorado State University, Fort Collins 80523, USA; Key Laboratory of Sustainable Forest Ecosystem Management-Ministry of Education, School of Forestry, Northeast Forestry University, Harbin 150040, China; Department of Land Surveying and Geo-Informatics, The Hong Kong Polytechnic University, Hong Kong 999077, China; Key Laboratory of Humid Subtropical Eco-Geographical Process (MOE), College of Geographic Sciences, Fujian Normal University, Fuzhou 350007, China; National Institute of Natural Hazards, Ministry of Emergency Management of China, Beijng 100085, China; National Institute of Natural Hazards, Ministry of Emergency Management of China, Beijng 100085, China; National Institute of Natural Hazards, Ministry of Emergency Management of China, Beijng 100085, China; National Institute of Natural Hazards, Ministry of Emergency Management of China, Beijng 100085, China; National Institute of Natural Hazards, Ministry of Emergency Management of China, Beijng 100085, China; Laboratory of Tree-Ring Research, University of Arizona, Tucson 85721, USA; Department of Land Surveying and Geo-Informatics, The Hong Kong Polytechnic University, Hong Kong 999077, China; National Institute of Natural Hazards, Ministry of Emergency Management of China, Beijng 100085, China; Department of Building Environment and Energy Engineering, The Hong Kong Polytechnic University, Hong Kong 999077, China; Laboratory of Tree-Ring Research, University of Arizona, Tucson 85721, USA

**Keywords:** fire–climate interactions, global warming, fire history network, urban fires, fire management

## Abstract

China, one of the most populous countries in the world, has suffered the highest number of natural disaster-related deaths from fire. On local scales, the main causes of urban fires are anthropogenic in nature. Yet, on regional to national scales, little is known about the indicators of large-scale co-varying urban fire activity in China. Here, we present the China Fire History Atlas (CFHA), which is based on 19 947 documentary records and represents fires in urban areas of China over the twentieth century (1901–1994). We found that temperature variability is a key indicator of urban fire activity in China, with warmer temperatures being correlated with more urban fires, and that this fire–temperature relationship is seasonally and regionally explicit. In the early twentieth century, however, the fire–temperature relationship was overruled by war-related fires in large urban areas. We further used the fire–temperature relationship and multiple emissions scenarios to project fire activity across China into the twenty-first century. Our projections show a distinct increase in future urban fire activity and fire-related economic loss. Our findings provide insights into fire–climate relationships in China for densely-populated areas and on policy-relevant time scales and they contribute spatial coverage to efforts to improve global fire models.

## Teaser

This study found that temperature variability is a key indicator of urban fire activity in China, with warmer temperatures are correlated with more urban fires, and that this fire-temperature relationship is seasonally and regionally explicit. In the early twentieth century, however, the fire-temperature relationship was overruled by war-related fires in large urban areas.

## INTRODUCTION

Fire is an earth system process that affects human communities and infrastructure, ecosystem structures and functions, and the local to global carbon cycle. It is an environmental process that signficantly impacts both the atmosphere and global climate [1–7], while weather conditions in turn shape the temporal and spatial attributes of regional fires [8–11]. Urban fires that occur in densely populated areas (including the city center and neighboring rural areas) have the most severe socioeconomic impacts. They destroy more property, create dangerous living conditions for more people, and increase the chance of human casualties compared to wildfires. In China, for instance, the most populated country in the world with the highest number of large cities, fire ranks first in natural disaster-related deaths, with more than 10 000 people [12,13], including residents and firefighters, dying in fires each year (1950–1994; Fig. S1). On local scales, factors such as occupant behavior, urban design, fire service performance, building code enforcement, and quality of home electrical products influence the occurrence of urban fires [11]. The mechanistic drivers of urban fire activity on larger scales, however, are largely unknown [14,15]. We know from wildfire–climate studies that rising temperatures and increasing droughts, combined in some regions with fuel accumulation due to long-term fire suppression, have contributed to the global increases in wildfire frequency and severity in late twentieth century [16–19]. The majority of such fire–climate studies, however, have focused on wildland fires and most studies—but not all [20–22]—are focused on North American landscapes. Urban fire regimes are more complex because of the manifold interactions with human activities. An improved understanding of the roles of climate and human activities on urban fires, as well as future trends in urban fire activity is essential for policy-makers to develop strategies to better control urban fires under future climate changes.

**Figure 1.**
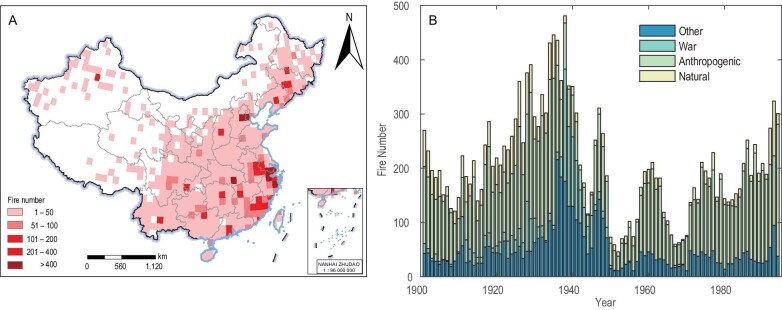
Spatial and temporal distribution of the China Fire History Atlas (CFHA). (A) Number of fires per 1° × 1° grid point in the CFHA (1904–1994), (B) total number of fires in the CFHA per year (1901–1994). Approval number: GS Beijing (2024) 0710.

To reveal the role of climate and anthropogenic factors in urban fires on large scales, we present a comprehensive temporally and spatially explicit database of the fire history of China in the twentieth century. We compiled 19 947 documentary fire records obtained from archives, libraries, and local chronicles from ∼660 cities in China [13], spanning 1901–1994 (see methods section; Fig. 1). Date, location, cause, and severity of each fire were recorded (see Supplemental Information, Photo S1). We then aggregated the individual fire data into a 1° × 1° gridded fire network—the China Fire History Atlas (CFHA). The CFHA mainly recorded significant fires with socioeconomic effects, and the routine crop residue burnings to enrich soil during the harvest seasons are not included. To validate CFHA, we extracted the urban fires from the Moderate Resolution Imaging Spectroradiometer (MODIS) fire product MOD14A2 (Supplemental Information). It confirmed that both spatial distribution patterns and seasonality are consistent between CFHA and MODIS fire products (Figs. S2 and S3), suggesting the robustness of our dataset.

## RESULTS

### Spatiotemporal variability of urban fires and their anthropogenic drivers

CFHA has a dense coverage over eastern China, particularly in the largest Yangtze River Delta City Group (e.g. Shanghai and Nanjing), the second largest Beijing–Tianjin–Hebei City Group, and the third largest Pearl River Delta City Group (e.g. Hong Kong and Guangzhou) (Fig. 1A). But the CFHA shows a poor coverage for western China (e.g. Tibetan Plateau and Xinjiang area) and part of Inner Mongolia where cities are sparsely distributed (Fig. 1A). The majority of fires in the CFHA are anthropogenic in nature (53% overall; Fig. S4A) and are caused by arson, accidents, etc. (Supplemental Information), and reflect the urban character of the CFHA. The fire time series for China shows more fires before than after 1949 (the foundation of the People's Republic of China) and a steady increase in the number of urban fires since 1970 (Fig. 1B). The high fire activity before 1949 occurred primarily in central China and large cities (Figs. S4 and S5) and is likely related to the frequent wars during that period. Indeed, peaks in the urban fire time series occurred during the Chinese Revolution of 1911, the Northern Expedition (1926–1928), World War Ⅱ (1931–1945), and the War of Liberation (1945–1949) (Fig. 1B and Fig. S6) [23]. More than half (53%) of the war-related fires were large or very large fires. Such war periods in China tended to be focused in large cities with high population density, where war caused urban fires directly, as well as through the associated social chaos that allowed robbers and bandits to burn and loot [13,23]. Limitations in fire-fighting capacity in residential areas might also have contributed to the high fire activity before 1949. Fire-fighting capacity improved after 1949, resulting in a decrease in urban fire activity, despite a rising population and urbanization in China starting in the 1950s (Fig. S7). The influence of increased fire-fighting capacity may have been limited in rural areas, however, where firefighters have to travel long distances to a fire and cannot reach it in a timely fashion.

The more recent increase in urban fires (1950–1994; Fig. 1B) can be related to China's rapid urbanization (*r* = 0.72, *p* < 0.001), economic growth (*r* = 0.66, *p* < 0.001), and population growth (*r* = 0.65, *p* < 0.001) [24], which resulted from the Chinese government's focus on Reform and Opening-up policy and Economic Growth [14]. The relative importance analysis indicated that the contribution of urbanization, economic growth, and population growth are all higher than that of climate factors (Fig. S8). With economic growth, urbanization and industrialization developed rapidly and substantial wealth was increasingly accumulated in cities [25]. With more and more fuels used in urban construction and consumption of electricity and fire, this led to an increased agglomeration of potential ignition sources [26]. At the same time, electrical failures due to overextension of the electric grid and aging wiring in super-scale and complex buildings induced increasingly frequent accidental fires. Every city in China still suffered from fire-fighting investment shortages during this period of economic transformation, and the fire safety level and ability to respond to emergencies was affected by the lack of public fire-fighting facilities [15].

**Figure 2.**
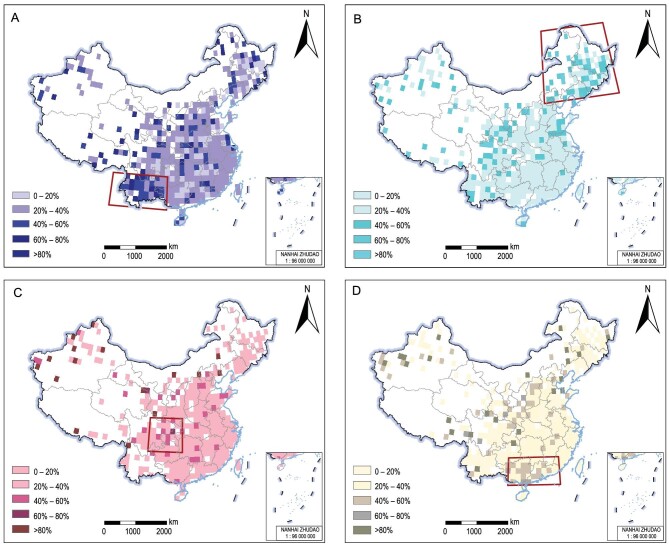
Fire seasonality maps of China. Percentage of fires (1901–1994) per grid point in the CFHA occurring in (A) winter (January–March), Southwest (96°–107°E, 21°–27°N), (B) spring (April–June), Northeast (115°–133°E, 40°–53°N), (C) summer (July–September), Central (103°–110°E, 27°–33°N), and (D) autumn (October–December), South (107°–117°E, 21°–26°N). Red frames indicate the region with the highest percentages for each season used for further analysis (Fig. 4 and Fig. S9). Approval number: GS Beijing (2024) 0710.

On a national scale, urban fires were evenly distributed seasonally, with fires common in winter (January–March: 28%), autumn (October–December: 27.4%), and spring (April–June: 26.4%), but less common in summer (July–September: 18.2%) (Fig. 2), in which fire season was defined by fire patterns in China (see Methods section). Fire seasonality varied strongly spatially (Fig. 2). Most winter fires occurred in southwestern China (e.g. Yunnan province; Fig. 2A), where winters are warm (13.9°C, absolute temperature from CRU TS 3.24) and dry (Fig. S9). Spring is the dominant fire season in northeastern China (Fig. 2B), where winters are generally cold and snow-covered and the climate during or before late spring is characterized by strong winds [20]. Fire risk is generally low in summer in China (Fig. 2C), due to the high precipitation associated with the summer monsoon. Yet, even under the wet conditions of the monsoon season (Fig. S9), fires still occurred in central China because of its hot climate (e.g. Chongqing City is one of the three hottest cities in China). Finally, fire activity in the subtropical areas of south China (e.g. Guangdong and Guangxi provinces) and west-central China is concentrated in autumn, a post-monsoon season with a relatively hot and dry climate (Fig. 2D). In addition to this, the duration of the fire season in the South is much longer than in northeastern China due to a lack of snowpack (Fig. S9). The spatial distribution of seasonal urban fires indicates the potential influence of climate conditions and suggests the need to identify potential climate indicators.

### Temperature as the key climate indicator of urban fires

**Figure 3.**
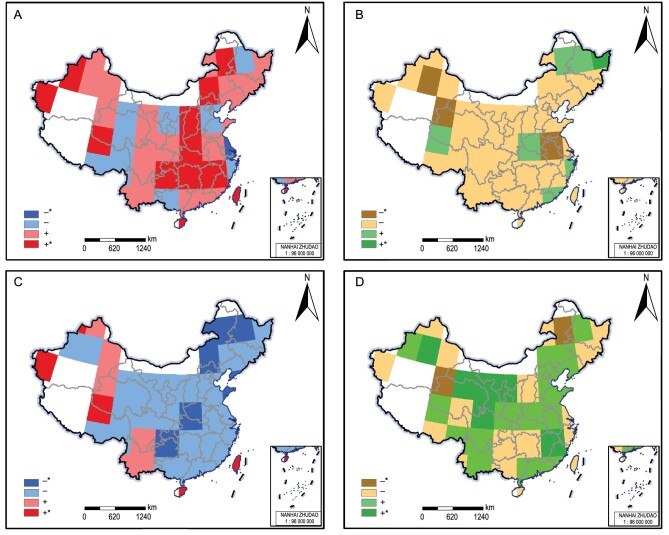
Climate anomalies during fire and non-fire years from 1901 to 1994 in the CFHA using the superposed epoch analysis method. Annual temperature (A and C) and SPEI (B and D) anomalies during the 20 largest fire years (A and B) and non-fire years (C and D) per 5° grid point in the CFHA (1901–1994). Blue/yellow colors indicate negative (cold or dry) anomalies, and red/green colors indicate positive (warm or wet) anomalies. Dark colors indicate statistical significance at the 95% level as identified based on a two-sample *t*-test. Approval number: GS Beijing (2024) 0710.

After excluding the effect of anthropogenic activities, temperature was the dominant climatic factor in urban fire variability, contributing much more than precipitation and drought (Fig. S8). In order to further identify the impacts of climate on extreme fire years, we composited temperature and drought conditions over the large fire years of the CFHA (Fig. 3 and Figs. S10 and S11). In more than 75% of the grid points, fire years were warmer and drier than average (Fig. 3A and B). The fire–climate relationship was statistically significant for nearly one-third (31%) of the grid points for temperature (Fig. 3A), but for only 7% of the grid points for drought (Fig. 3B). The fire–drought relationship, however, was more spatially robust, with fire years in more than 83% of the grid points occurring during dry years. Low-fire years, on the other hand, were generally cooler and wetter than normal (Fig. 3C and D). This relationship was most spatially robust for temperature, with 74% of the grid points showing cold conditions during low-fire years, and was most pronounced in the northeastern region, where 50% of the grid points showed significant results. Composite maps of the 1° × 1° and 2° × 2° networks had similar patterns (Figs. S10 and S11).

We compiled all fire years, not only the extreme years, in one time series (1901–1994) and produced the China Fire Chronology (CFC). The CFC is composed of standardized time series (see Methods section), thus diminishing the contribution of a few large cities with high fire numbers in the composite time series. A fire–climate correlation analysis based on the CFC showed that temperature variability trumped moisture variability in its influence on urban fire activity (Fig. S12). During 1901–1994, the CFC was strongly significantly positively correlated with mean annual temperature (MAT) (*r* = 0.58, *p* < 0.001), but we did not find strong correlations with precipitation (*r* = 0.004, *p* > 0.1) or drought indices (Palmer Drought Severity Index (PDSI), *r* = 0.12, *p* > 0.1; Standardized Precipitation Evaporation Index (SPEI), *r* = 0.28, *p* < 0.01). To verify that the fire–temperature relationship was not influenced by a drastic change in the number of meteorological stations after 1949 [27], we calculated the CFC–MAT relationship for the post-1950 period separately and found a similarly strong significant positive correlation (*r* = 0.57, *p* < 0.001, 1950–1994). We further verified that the CFC–MAT relationship was not solely driven by a simultaneous—but not necessarily related—upward trend in both records, for example, due to a simultaneous temperature and population increase. For this purpose, we high-pass filtered (*f* > 0.05) the CFC and MAT time series and found that CFC and MAT were also positively related at the interannual scale (*r* = 0.41, *p* < 0.001, 1901–1994). The correlations between fires and the minimum and maximum temperatures were similar to the mean temperatures (Table S1). By contrast, we found only a weak correlation between CFC and population size (raw data, *r* = 0.56, *p* < 0.001, 1901–1994; high-pass filtered data (*f* > 0.05), *r* = 0.22, *p* < 0.05, 1901–1994).

We also found a significantly positive correlation (*r* = 0.25, *p* < 0.05, 1901–1994; Table 1) when comparing the total (non-standardized) annual number of fires in China (Fig. 1B) to MAT. This correlation is stronger for the more recent half of the period (*r* = 0.57, *p* < 0.01, 1950–1994) compared to the first half (*r* = 0.25, *p* < 0.1, 1901–1950). To test whether the fire–temperature relationships are influenced by the cities with a particularly high number of fires, we compared temperature with the number of fires in high-fire-number areas (31 grid points with more than 100 fire activities for each grid, namely large cities) versus fires in low-fire-number areas (551 grid points with less than 100 fire activities, namely small cities). We found that the relationship with fires in low-fire-number areas is consistently strong through time (Table 1), whereas in high-fire-number areas it is only significant after 1950 (*r* = 0.52, *p* < 0.01, 1951–1994). This confirms that high-fire-number areas in the early twentieth century, particularly in the 1930s (Fig. 1B and Fig. S5), were likely caused by wars, rather than by warm temperatures. Large urban areas were the first to bear the brunt of these wars [23] (Figs. S4 and S6) and their fire–temperature relationship was likely overruled by war-related fires. We also used long meteorological data records to test the climate–fire relationships for six big cities (Beijing, Shanghai, Guangzhou, Taiyuan, Xiamen, Wenzhou) with both continuous meteorological and fire records, the results are similar to using CRU climate data (Table S2). In order to clarify the effect of temperature on urban fires with four causes, we tested the relationship between fire and temperature and found that anthropogenic fires were significantly correlated with MAT (*r* = 0.31, *p* < 0.01), stronger than the correlation between MAT and the other three types of fires, proving that temperature was associated with urban fires indirectly through its influence on human activities. We additionally tested the fire–temperature relationships for six fire severity levels and found that the positive correlation is particularly robust through time for large fires (Table 1). On the other hand, the fire–temperature relationship for small and medium fires is only significant in recent decades (*r* = 0.33, *p* < 0.05, 1951–1994; Table 1).

**Table 1.** The relationship between fire and climate in China for three periods in the twentieth century. Pearson correlation coefficients between mean annual temperature and number of fires in China.

	1901–1950	1951–1994	1901–1994
All fires	0.25*	0.57***	0.25**
Super, very large and large fires	0.57***	0.60***	0.49***
Medium and small fires	0.08	0.33**	0.04
High-fire-number areas	0.06	0.52***	0.10
Low-fire-number areas	0.57***	0.57***	0.59***

*p < 0.1, **p < 0.05, ***p < 0.001

The positive response of urban fire activity to temperature is in line with the increase in wildfires under a warming trend in China [28], as well as globally [10,16,29]. One difference is that wildland fires often show strong responses to both temperature and drought [30,31], whereas our results suggest that temperature is the main climate indicator of urban fires. The influence of drought on urban fires is weak and mostly non-significant (Fig. 3B). This can be explained by the difference in nature and moisture content of combustible materials in forests versus urban areas [32]. The fuel load of wildland fires consists primarily of vegetation, the moisture content of which is influenced by both soil moisture content and evapotranspiration [16,33]. Dry conditions decrease soil moisture and as such decrease the moisture content of vegetation and dead fuels. The situation is different for urban fires, where combustible materials consist primarily of construction materials—even if more and more reinforced concrete material is used in urban construction after the reform and opening-up policy since 1978—the moisture content of which is also influenced by evaporation, but not by soil moisture. Urban combustible materials thus dry out and become more combustible with warmer temperatures and their moisture content is thus more determined by temperature. In addition to this, temperature not only modulates combustibility but may also impact urban fires through modulating anthropogenic activities. There is also growing evidence that people tend to be more impatient and violent under warmer conditions [34–36] and ignition and arson are thus more likely to occur. More people working outside with more chances for accidental ignitions (or purposeful that get away from them, such as welding or burning trash), overstressed electrical grids and sparking, etc. (just more chance of ignitions during warmer conditions with more people and more outdoor activities).

**Figure 4.**
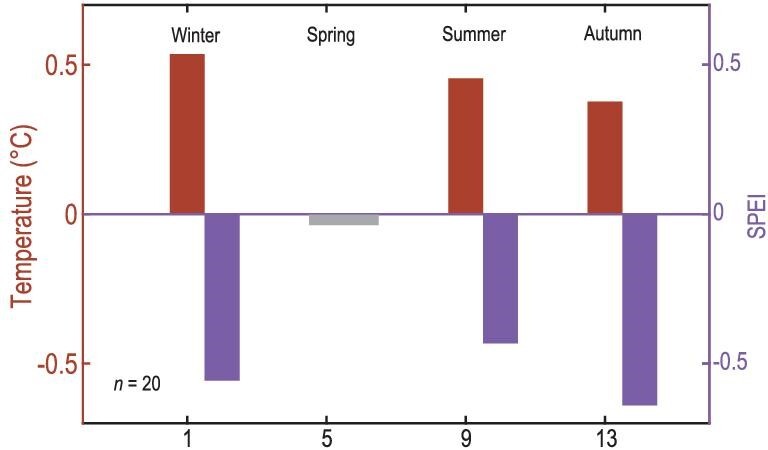
Climate anomalies during fire years from 1901 to 1994 in four subregions of China using the superposed epoch analysis method. Temperature (red) and SPEI (purple) anomalies determined by a composite analysis of the 20 largest fire years in four subregions of China, indicated by red boxes in Fig. 2. Southwest (96°–107°E, 21°–27°N), Northeast (115°–133°E, 40°–53°N), Central (103°–110°E, 27°–33°N) and South (107°–117°E, 21°–26°N). Red/purple bars mark statistically significant departures (*p* < 0.05) from mean conditions.

The increase in urban fire activity with warmer temperatures we find for China is consistent with fire–temperature relationships in other low-latitude regions, such as Australia [37] and Indonesia [11]. At higher latitudes, such as Great Britain [38] and the Northeastern US [39], however, the urban fire–temperature relationship can be reversed, with more fire activity in cold conditions. This is particularly the case in cold winters, when more fire accidents occur due to space heaters, electric blankets, and faulty electricity lines. We found generally positive relationships between fire activity and temperature throughout China, regardless of the season, latitude, or region (Fig. 3 and 4). In the three low-latitude regions that show a distinct seasonality in their fire activity (Fig. 2; southwestern, southern, and central China), we found that fires typically occur during warm and dry seasons and conditions (Fig. 3 and 4 and Fig. S9), which is also when wildland fires occur in these regions [40,41]. In high-latitude northeastern China, urban fires are most prevalent in spring, which also is a relatively warm and dry season there [20,42,43] (Fig. S9), but were only weakly related to regional temperature or drought variability (Fig. 4 and Fig. S13).

### Twenty-first century projections of fire activity and economic loss in China

**Figure 5.**
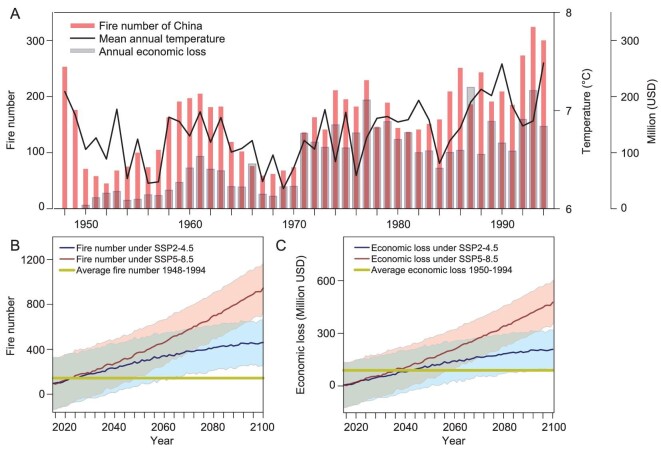
Historical and projected number of fires, mean annual temperature, and fire-related economic loss in China. (A) Number of fires, mean annual temperature (1948–1994), and fire-related economic loss in China (1950–1994); projected (2015–2100) annual number of fires (B) and annual economic loss by fire (C) in China under two shared socioeconomic pathways (SSPs). The green line represents the average annual number of fires (1948–1994) (B) and average annual economic loss (1950–1994) (C). The shaded areas represent standard deviations of projected annual number of fires (B) and annual economic loss (C). The economic loss from 1950 to 1994 was transformed through the year-by-year exchange rate of RMB to USD. The projected economic loss from 2015 to 2100 was transformed through the average exchange rate of RMB to USD in 2023.

Post-1950 urban fire activity in China has evolved in synchronization with urbanization and economic growth, but our analysis shows that at large scales, interannual temperature variability, as well as a long-term warming trend, also played an important role. The evolution of urban fire activity thus also closely matches decadal-scale temperature variability, as demonstrated by low fire activity during the relatively cool 1950s and a steady increase in fire activity since the early 1970s, in synchronization with rising temperatures (Figs. 1B and S12). Satellite records suggest that, coinciding with a global warming trend, the late-twentieth-century increase in fire activity continued into the early twenty-first century (2001–2016) [44]. In addition to this, fire activity and independently derived fire-related economic loss in China are strongly and positively correlated (*r* = 0.77, *p* < 0.001, 1950–1994, Table S3). As a consequence, fire-related economic loss and MAT over China are also strongly positively correlated (*r* = 0.57, *p* < 0.001, 1950–1994, Table S3). The average annual economic loss caused by fire in China from 1950 to 1994 was RMB272.4 million (∼US$32 million) (Fig. 5A).

Based on the post-1950 relationship between MAT and the total annual number of fires in China (Fig. 5A and Table 1), we established a linear regression model to project urban fire activity into the twenty-first century using MAT projections from the Coupled Model Intercomparison Project Phase 6 (CMIP6) [45] model output. Under the Fossil-fueled Development SSP5-8.5 scenario, annual fire activity in China from 2015 to 2040 was expected to increase by 4%–16% compared to the period 1948–1994 (Fig. 5B). However, from 2041 to 2100, the ensemble simulations predicted annual fire numbers up to three times the average between 1948 and 1994. Under the Middle of the Road SSP2-4.5 scenario, annual fire activity in China was expected to grow more slowly by 2100, but still increase significantly (Fig. 5B). The occurrence of urban fires is closely related to economic loss, so we predicted fire-related economic loss in the twenty-first century based on the fire–MAT regression relationship (Fig. 5A and Table S3). The result showed that the average annual economic loss from 2015 to 2100 was expected to be 3.1 times (RMB900 million or US$128 million under SSP2-4.5) to 5.6 times (RMB1.5 billion or US$213 million under SSP5-8.5) than the average annual loss from 1950 to 1994 (Fig. 5C). Notably, annual economic loss under the SSP2-4.5 scenario was expected to rise to more than RMB1.5 billion (US$213 million) by the end of this century, while annual economic loss under the SSP5-8.5 scenario was expected to rise to RMB3.4 billion (US$482 million). The result of the relationship between temperature and fire addresses that the rise of temperature and the increase of fire activity in China would affect many human beings, accompanied by a severe economic impact and ecological damage.

## DISCUSSION AND CONCLUSIONS

The CFHA is unique in its national scale coverage over a previously unstudied region and complements a growing global-scale network of fire history data while at the same time increasing the spatial coverage across East Asia. We found that temperature is the dominant climate indicator of urban fires in China and our projections suggest a dramatic twenty-first-century increase in urban fires and related economic loss. With ongoing public concern regarding rising temperatures affecting fire activity and its risk, our datasets are critical for understanding the impacts of climate change on urban fires in China and our results provide a direct data contribution to improve global fire models.

Prior to the foundation of the People's Republic of China in 1949, we found that wars also played an important role, particularly in large cities. Since 1949, urbanization, economic growth, and population growth have accelerated the rate of urban fires, while the rapid development of fire-fighting capacity and continuous improvement of the fire-fighting system [46] has suppressed the intensity of urban fires. The contribution of these factors on urban fires is nonlinear, and it is uncertain which contribution is higher, indicating that human activities have actually a double-edged effect when it comes to fire.

Our study identifies regional fire seasons in China, which can aid fire managers in identifying high fire risk areas and in seasonally concentrating suppression efforts [47], while better communicating the complexities of fire management to the general public [48–50]. Urban and wildland fire agencies will thus need to cooperate in fire prevention, such as focusing on the rapid growth of the wildland–urban interface [51–53]. The tangible examples can be understood by the public and a broad scientific audience, and can potentially have positive effects on climate policy [54–56].

In recent years, China has witnessed a series of severe urban fire incidents, leading to substantial loss of life and property. It is imperative that the central government needs to emphasize the financial investment in urban fire prevention strategies, enhancement of infrastructure resilience, expansion of funding for scientific research in this domain, and raise public awareness about fire prevention.

Although this study presents the relationship between urban fires and climate and indicates the influence of other factors on urban fires in China, it also identifies areas in need of improvement. (1) Due to data limitations, our analysis is not extended beyond 1994. Future research could incorporate multisource satellite data, human activity data, and detailed case studies of fires to analyze the driving factors of urban fires [57,58]; (2) by integrating data on wildfires, the study could confirm the impact of climate on the synergistic effects of wildfires and urban fires; (3) in conjunction with the distribution of firefighters and resource allocation, detailed zoning maps for urban and wildland fires in China could be developed. These efforts will be helpful to mitigate the economic and human losses caused by fires.

## METHODS

### Fire data

We compiled fire data from a national fire survey conducted in the 1990s, in which more than 5 000 researchers and over 50 000 archives and libraries were involved [13]. The fire dataset consists of more than 36 000 documentary data entries that include the date, location, cause, and severity of individual fires in China. The records cover the period 4300 BCE–1994 CE with more than half of the data entries (19 947 records) recording twentieth-century fire activity and we have restricted our analysis to these 94 years (1901–1994) with consistent data coverage. Over this period, fires were categorized by one of six severity levels (super: 1%, very large: 17%, large: 18%, medium: 25%, small: 33%, unknown: 6%; Fig. S14). The definition of fire severity categories was based on the China Fire Standards [59]. Detailed descriptions of the severity categories, as well as how we dealt with exceptional cases, are provided in the Supplemental Information, section ‘Definition of fire severity levels.’

We classified the fire data as occurring in one of four seasons: winter (January–March), spring (April–June), summer (July–September), and autumn (October–December) and then aggregated the seasonal data into a 1° × 1° gridded network [27] that covers a large portion of China with 582 grid points (Fig. 1A). The seasonal distribution of urban fire numbers does not follow the traditional or monsoon-based definition of seasons (Fig. S15). Instead, fire numbers are particularly high from January to March, decrease from April to June, and rebound from July to December (Fig. S16). Accordingly, we define our fire-related seasons based on those fire patterns, which are very similar to the traditional season definitions, but with a one-month lag.

### Climate data and composite analysis

To spatially identify the climate indicator of fire in China, we applied a composite analysis to the CFHA to evaluate the relationship between fire activities and annual temperature, precipitation, and drought indices (Standardized Precipitation Evapotranspiration Index (SPEI); Palmer Drought Severity Index (PDSI)) at the grid point level (Figs S10 and S11). We derived gridded climate data from the CRU TS 3.24 dataset (temperature and precipitation) [60], from the CSIC SPEI Drought Index dataset (SPEI) [61], and the CRU scPDSI 3.25 dataset (scPDSI) [62]. We have aggregated the PDSI and SPEI data on a regular 0.5° grid to a 1° grid to facilitate comparisons with datasets of fire, temperature, and precipitation that were available on a regular 1° grid.

We eliminated from the analysis 32 (out of 582) grid points that entirely missed climate data (SPEI and PDSI). For the remaining grid points, we filled occasional missing values in the climate dataset with monthly means. From the remaining 550 grid points, we selected only those grid points that had at least 5 fire years—defined as years with at least one recorded fire—over the 94 years of analysis. This selection criterion eliminated an additional 212 1° grid points from our analysis. For the remaining 338 grid points, we selected the 20 largest fire years (years with the highest number of fires) and used them as event years in a composite analysis with the four climate parameters (Fig. S10). For grid points with more than 5 but less than 20 fire years, we composited overall fire years. The significance of differences in composite climatic conditions between the largest fire years and all other years was identified using a two-sample *t*-test.

To circumvent elimination of a large number of grid points due to missing values and low numbers of fire years, and to investigate the robustness of fire–climate relations at a broader scale, we also aggregated the 1° × 1° gridded data into 2° × 2° (Fig. S11) and 5° × 5° grids (Fig. 3). For this purpose, we summed the fire data for every 4 (or 25) adjacent 1° grid points to form a 2° (or 5°) grid point. Each of the new 2° (or 5°) grid points contained at least one record in its original 4 (or 25) adjacent 1° grid points. We selected from the 2° × 2° and 5° × 5° grids only those grid points that had at least 5 fire years over the 94 years of analysis. This resulted in a network of 144 2° grid points and 42 5° grid points, for which the 20 largest fire years were then used in a composite analysis (Fig. S11 and Fig. 3). The 5° × 5° fire grid also allowed us to composite climate variables over low-fire years (Fig. 3C and D), defined as the 20 years with the lowest number of fires. The 1° × 1° and 2° × 2° networks had too many non-fire years per grid point for this analysis. To aggregate the corresponding 2° and 5° grids for the four climate datasets, we had to include some 1° grid points with missing values. In this case, the values of all 1° grid points within each 2° and 5° grid point were summed. The data analysis framework diagram is presented in Fig. S17.

### Temporal analysis

To study the overall effect of climate variability on fire in China in the twentieth century and to project fire–climate relationships into the twenty-first century, we combined the 582 1° grid point time series of the CFHA into a single time series. For this purpose, we first standardized the time series for each grid point (giving all grid points the same mean (0) and standard deviation (1)) and then averaged these 582 time series into one CFC (Fig. S12). The CFC puts relatively low weight on the few large cities with high fire numbers and thus gives an overview of urban fires in the whole of China. We also calculated seasonal CFC time series by averaging the standardized seasonal (winter, spring, summer, and autumn) time series for grid points in each of the four subregions where fire seasonality was strongest (Fig. 2). For instance, we created a winter fire chronology by averaging the standardized winter fire time series for the southwestern China region in Fig. 2A. Finally, to study fire seasonality and to investigate seasonal fire–climate relationships, we composited seasonal regional temperature and SPEI values for the 20 largest fire seasons (winters, springs, summers, and autumns with the highest fire number) in each of the four subregions where fire seasonality was strongest (Fig. 4).

To investigate the influence of temperature on fire activity and fire-related economic loss in China, we complemented the fire records with fire-related annual economic loss data, independently derived from the Fire Statistical Yearbook of China [12] (Fig. 5A; 1950–1994). We correlated the CFC time series (1901–1994) and the fire-related economic loss time series (1950–1994) with a mean annual temperature (MAT) time series. The MAT time series was calculated as the average of the MAT values of the CRU TS 3.24 1° grid points that fall within China's land boundaries and cover the entire country.

To project fire activity and economic loss in China into the twenty-first century, we calculated the total annual number of fires per year for the entire country (Fig. 1B). The total annual number of fires is a direct measure of the severity of fires of the country relative to the CFC, which was standardized to have equal weight among cities. We then used the post-1950 relationship between this annual number of fires, economic loss, and MAT to create a simple linear regression model and to project fire activity and loss in China into the twenty-first century using MAT projections from the Coupled Model Intercomparison Project Phase 6 (CMIP6) model output [45]. CMIP6 includes simulations from 13 climate modeling organizations (Table S4) and we used the modeled temperatures for the period 2015 to 2100. CMIP6 includes eight Shared Socioeconomic Pathways (SSPs) based on eight greenhouse gas concentration trajectories: SSP1-1.9, SSP1-2.6, SSP2-4.5, SSP3-7.0, SSP4-3.4, SSP4-6.0, SSP5-3.4, and SSP5-8.5. We selected simulated MAT based on two of these trajectories (SSP2-4.5 and SSP5-8.5). We utilized the 48 models to obtain 48 model projections of the annual fire number and fire-related economic loss. To reflect the deviation between model outputs, we calculated averages and standard deviations of the MAT, fire number, and fire-related economic loss across the 13 modeling groups (Fig. 5B and C).

## Supplementary Material

nwae163_Supplemental_File

## DATA AND MATERIALS AVAILABILITY

All data are available in the Supplemental Information.
