# Supplementary material for: Anthropogenic warming is a key climate indicator of rising urban fire activity in China
Source: Natl Sci Rev. 2024 May 7;11(5):nwae163. doi: 10.1093/nsr/nwae163 (PMC11162151; doi:10.1093/nsr/nwae163)
Supplement: nwae163_Supplemental_File [file nwae163_supplemental_file.doc]

Supplemental Information for

**Anthropogenic warming is a key climate indicator of the rising urban fire activity in China**

Qichao Yao^a, b, c^, Dabang Jiang^d, 1^, Ben Zheng^e^, Xiaochun Wang^b, 1^, Xiaolin Zhu^f^, Keyan Fang^g^, Lamei Shi^a^, Zhou Wang^a^, Yongli Wang^a^, Linhao Zhong^a^, Yanyan Pei^a^, Amy Hudson^c^, Shuai Xu^f^, Maowei Bai^a^, Xinyan Huang^h^, and Valerie Trouet^c, 1^

^1^ Correspondence to: [jiangdb@mail.iap.ac.cn](mailto:jiangdb@mail.iap.ac.cn), wangx@nefu.edu.cn, [trouet@ltrr.arizona.edu](mailto:trouet@ltrr.arizona.edu)

**This PDF file includes:**

Supplemental Information: **Definition of fire cause and severity levels**

**Contents for the data quality control**

References for SI reference citations

**Fig. S1.** Twentieth century trends in fire-related casualties in China.

**Fig. S2.** The spatial distribution of normalized fire frequency of CFHA and MODIS data.

**Fig. S3.** Fire seasonality map of China (2001–2005) derived from MODIS fire product.

**Fig. S4.** Cause of fires in China.

**Fig. S5.** Fire severity maps of China.

**Fig. S6.** Twentieth century fire chronologies in China.

**Fig. S7.** Twentieth century fire chronologies in large and small cities in China.

**Fig. S8.** Fire number and population in China (1901–1994).

**Fig. S9.** Seasonal climate variability for four subregions of China.

**Fig. S10.** Climate anomalies during fire years across China (1° × 1°).

**Fig. S11.** Climate anomalies during fire years across China (2° × 2°).

**Fig. S12.** Z-scores of China fire chronology (CFC), mean annual temperature, fire-related annual economic loss, and annual precipitation days.

**Fig. S13.** Annual and seasonal climate anomalies during fire years in Northeast China.

**Fig. S14.** Distribution of fire percentages in the China Fire Historical Atlas (CFHA) following the monsoon-based seasonal definition.

**Fig. S15.** Monthly distribution of the percentages of urban fires during the period of 1901–1994.

**Fig. S16.** Relative importance of urbanization, economic growth, population growth and climate factors (MAT, precipitation and scPDSI) on fire activity.

**Fig. S17.** Framework diagram of data analysis.

**Table S1.** Pearson correlations of the annual CFC and fire number with the maximum, minimum and mean temperature.

**Table S2.** Pearson correlation coefficients between the first-order differenced mean annual temperature and number of fires in six large cities.

**Table S3.** Fire, temperature and fire-related economic loss interaction.

**Table S4** Projection models.

**Photo S1.** Examples about the fire descriptions in documentary records.

**Captions for Movie S1.** The China Fire History Atlas.

**Supplemental Information**

**Definition of fire cause and severity levels**

***The following definitions of fire cause and severity categories are based on China Fire Standards* (**1, 2**)**

***Fire cause (number means reasons)***

1. Anthropogenic: intentional arson, cigarette embers, careless accidents, kitchen accidents, play with fire, firework, firecracker, incense (Chinese customs), burn paper (Chinese customs), welding, baking, chemical accident, transport and storage of dangerous goods.
2. Natural: lightning, spontaneous combustion, earthquake, comet impact and unknown impact, explosion, leakage of electricity (non-subjective).
3. War: intentional war arson, secret arson.
4. Other: non-detailed description or unknown.

***Fire severity levels (number means severity levels)***

-1- Super Fire: Fire that caused more than RMB100,000,000 (US$11,602,678, using the average exchange rate of RMB to USD in 1994, 0.1160) in property damage or more than 1,000 houses burnt down.

0- Very Large Fire: Fire that caused any (or multiple) of the following: (1) 10 or more casualties, (2) 20 or more serious injuries, (3) 20 or more casualties or serious injuries, (4) 50 or more houses affected, (5) more than RMB1,000,000 (US$116,027) in property damage.

1- Large Fire: Fire that caused any (or multiple) of the following: (1) 3 to 9 casualties, (2) 10 to 19 serious injuries, (3) 10 to 19 casualties or serious injuries, (4) 30 to 49 houses affected, (5) RMB300,000 (US$34,808)–RMB1,000,000 (US$116,027) in property damage.

2- Medium Fire: Fire that caused any (or multiple) of the following: (1) 1 or 2 casualties, (2) 3 to 9 serious injuries, (3) 3 to 9 casualties or serious injuries, (4) 10 to 29 houses affected, (5) RMB100,000 (US$11,603)–RMB300,000 (US$34,808) in property damage.

3- Small Fire: Fire that caused (1) no casualties, (2) less than 3 serious injuries, (3) less than 10 houses affected, (5) less than RMB100,000 (US$11,603) in property damage.

4- Unknown: No specific description of fire; boat fire and no description of the property damage.

These are the levels that the fire cause and severity categories are based on. Categories are determined by categorizing the available information in this order: (1) determine if there is specific property damage information; if none then (2) determine if there is information about the number of affected houses; if none then (3) determine if there is information about casualties or serious injuries. If none of the information mentioned above is available, then categorize the fire as a small fire. However, exceptional cases have occurred and below we summarize how we have proceeded with these exceptions.

***Rules applied in categorizing exceptional fire cause cases.***

a- If the fire was set by bandits, soldiers, Japanese army, Fani (disparaging name for rebel in Qing Dynasty), etc., categorize the fire cause as war.

b- If ‘Zei (e.g. thief)’ in records means thieves, categorize the fire cause as anthropogenic; if ‘Zei (e.g. thief)’ means soldiers, categorize the fire cause as war.

c- If there are records such as ‘the causes are unknown’ or ‘the casualties are unknown’, categorize the fire cause as other.

***Rules applied in categorizing exceptional fire severity cases.***

a- Three rooms are regarded as one house.

b- Statue burned: medium fire.

c- Fire can be seen from ‘10 Li’ (5 kilometers) away: super fire.

d- If the records involve ‘Gui Chao’ (a currency once used in Guangxi Province), for instance, ‘loss of four thousand Gui Chao’, then the fire cannot be categorized by property damage.

e- The whole street burnt down – very large fire.

f- If the village is burnt down, categorize the fire by the number of deaths as super fire or very large fire.

g- If oil or other fuel is mentioned, categorize fire as super fire or very large fire.

h- If several fires are recorded in the same place and at the same time, categorize the fire by the total number of burnt houses or total property damage.

i- If smoke suddenly appears and is blown in the wind, categorize the fire as small fire.

j- For records such as ‘one hundred Liang of golden leaves, phoenix coronet’, ‘ten thousand Liang of silver’, etc., categorize the fire as large fire or larger.

k- For records such as ‘all houses are burned down, which is a great disaster’, ‘fallacies are spread to mislead people which lead to great disasters’, categorize the fire as super fire or very large fire accordingly.

**Contents for the data quality control:**

**Background**

The collection of the CFHA dataset cost massive amounts of labor and time. More than 5,000 researchers and over 50,000 archives and libraries were involved. The CFHA dataset has abundant details about urban fires. However, it is necessary to assess its quality with independent datasets. Satellites provide repeated observation over the whole Earth’s surface, so they have been explored to detect fires on land surface and achieved satisfactory results (3–8). Satellite fire products cannot replace the CFHA data in this study, because they have short records and they cannot provide information such as urban fire cause and severity, but they can be used to assess the quality of the CFHA data.

**Data**

In the study, the Moderate Resolution Imaging Spectroradiometer (MODIS) fire product, MOD14A2, was selected to assess the quality of CFHA data because (a) the MODIS has a daily revisit cycle and is suitable to detect urban fire activities that are sudden events and detectable only over a short period; and (b) the advanced MODIS active fire detection algorithm can obtain accurate results with an error lower than 1.2% (3). The MOD14A2 fire data are generated at 1 km spatial resolution. The MOD14A2 gridded composite contains the maximum value of the individual fire pixel classes detected during the eight days of acquisition. The MOD14A2 is appropriate to derive urban fire data because it records the daily fires with the highest confidence in each eight days.

**Methods**

The MOD14A2 includes all types of active fires on land surface, including wildfires and urban fires. To extract urban fires from the MOD14A2 product, we implemented the following steps to process the original MOD14A2 product.

**Step 1:** mosaic all the MOD14A2 images over China within the 2001–2005 period. The selection of this period gives us five years of fire data that can be compared to the CFHA. The MODIS data are available from February 18, 2000 onwards, and the first full year of data is thus 2001.

**Step 2:** generate the urban mask from the MODIS land cover product (MCD12Q1) (9). The CFHA dataset only contains fires in rural and urban areas rather than wildfires, and it is necessary to extract urban fires from the original MODIS fire data to make it consistent with the CFHA data. From the MODIS land cover product, pixels of urban and build-up class were extracted as urban areas. In addition, considering that rural areas around urban areas are included in the CFHA dataset, a 5-by-5 moving window was used to identify potential rural areas, i.e., if the moving window has urban pixels and its central pixel is not forest land, the central pixel was then marked as a rural pixel. Then, only fires in urban and rural pixels were kept from the original MODIS fire product.

**Step 3:** aggregate the MODIS urban fire data into a 1° × 1° grid, the same spatial unit as used for the CFHA data. When aggregating the 1 km MODIS fire product to 1° × 1° grid points, only the fire pixels with high confidence (i.e., pixel value = 9 in the MOD14A2 product) were counted to reduce uncertainty in the MODIS fire product.

We then compare the consistency of spatial distribution patterns and seasonality of urban fires over 5 years between the two datasets. We selected the most recent 5 years (1990–1994) of the CFHA dataset and the earliest 5 years (2001–2005) of the MODIS dataset for comparison.

To assess the consistency of urban fire spatial distribution patterns between these two datasets that use different ways to detect fires and cover different periods, the datasets need to be normalized to the same value range. Specifically, we first implemented a log transformation to both datasets to make the strongly skewed distributions (i.e., there are many more grid cells with small number of fires than large number of fires) less skewed. Then, the log-transformed data were rescaled to the 0–1 by the min-max normalization approach. Moran’s I index (10) was computed for the rescaled fire frequency to evaluate the spatial dependence of both datasets. Finally, the rescaled fire frequencies of both datasets were mapped for visual comparison and correlation coefficients between these two datasets were calculated to evaluate the consistency.

To assess the similarity of seasonality between the two datasets, the urban fires extracted from the MODIS fire product (2001–2005) were summarized into seasonal percentages (i.e., 100% × number of fires in each season / total number of fires) for each grid. Then, this seasonal percentage was mapped and compared with those from the CFHA data.

**Results**

**Fig. S2** shows the maps of the rescaled fire frequency from both datasets. In these maps, grids with 0 indicate no fires happened and grids with 1 have the maximum fire frequency. We can see that the spatial patterns of these two maps correspond well with each other. In other words, the highest number of urban fires happen in the highly populated areas in both datasets. The spatial patterns of these two datasets are significantly correlated (*r* = 0.37, N = 1115, *p* < 0.001). Fire frequency from both datasets shows similar positive spatial autocorrelation (Moran’s I values are 0.47 and 0.66, respectively).

**Fig. S3** shows the seasonal percentage of urban fires from the MODIS dataset. Compared with the seasonality maps from the CFHA data (Fig. 2), the urban fires from MODIS data show similar seasonality. For example, both datasets show most October–December fires happened in southeast China, and many January–March fires occurred in Yunnan province.

Above assessments using the MODIS product as independent validation data suggest that the CFHA data are a reliable dataset.

**References**

1. State Council of China. *No. 493 Decree of the State Council of China: Handling rules of production safety accident report and investigation.* https://www.gov.cn/gongbao/content/2007/content_632082.htm (10 September 2023, date last accessed).
2. Ministry of Public Security of China, Ministry of Labor of China and National Bureau of Statistics of China. *No. 82 Decree of the Ministry of Public Security of China: Fire statistics management regulations*. https://www.gov.cn/gongbao/content/2006/content_421771.htm (10 September 2023, date last accessed).
3. Giglio L, Schroeder W and Justice C O. The collection 6 MODIS active fire detection algorithm and fire products. *Remote Sens Environ* 2016; **178**: 31–41.
4. Giglio L, Loboda T V and Roy D P *et al*. An active-fire based burned area mapping algorithm for the MODIS sensor. *Remote Sens Environ* 2009; **113**: 408–420.

5. Giglio L, Kendall J D and Justice C O. Evaluation of global fire detection algorithms using simulated AVHRR infrared data. *Int J Remote Sens* 1999; **20**: 1947–1985.

6. Li Z, Nadon S and Cihlar J. Satellite-based detection of Canadian Boreal forest fires: Development and application of the algorithm. *Int J Remote Sens* 2000; **21**: 3057–3069.

7. Li Z, Kaufman Y J and Ichoku C *et al*. A review of AVHRR-based active fire detection algorithms: Principles, limitations, and recommendations. *Int J Remote Sensing* 1999.

8. Schroeder W, Oliva P and Giglio L *et al*. Active fire detection using Landsat-8/OLI data. *Remote Sens Environ* 2016; **185**: 210–220.

1. Friedl MA, Sulla-Menashe D and Tan B *et al*. MODIS Collection 5 global land cover: Algorithm refinements and characterization of new datasets. *Remote Sens Environ* 2010; **114**: 168–182.

10. Li H, Calder CA and Cressie N. Beyond Moran’s I: Testing for spatial dependence based on the spatial autoregressive model. Geogr Anal 2007; **39**: 357–375.


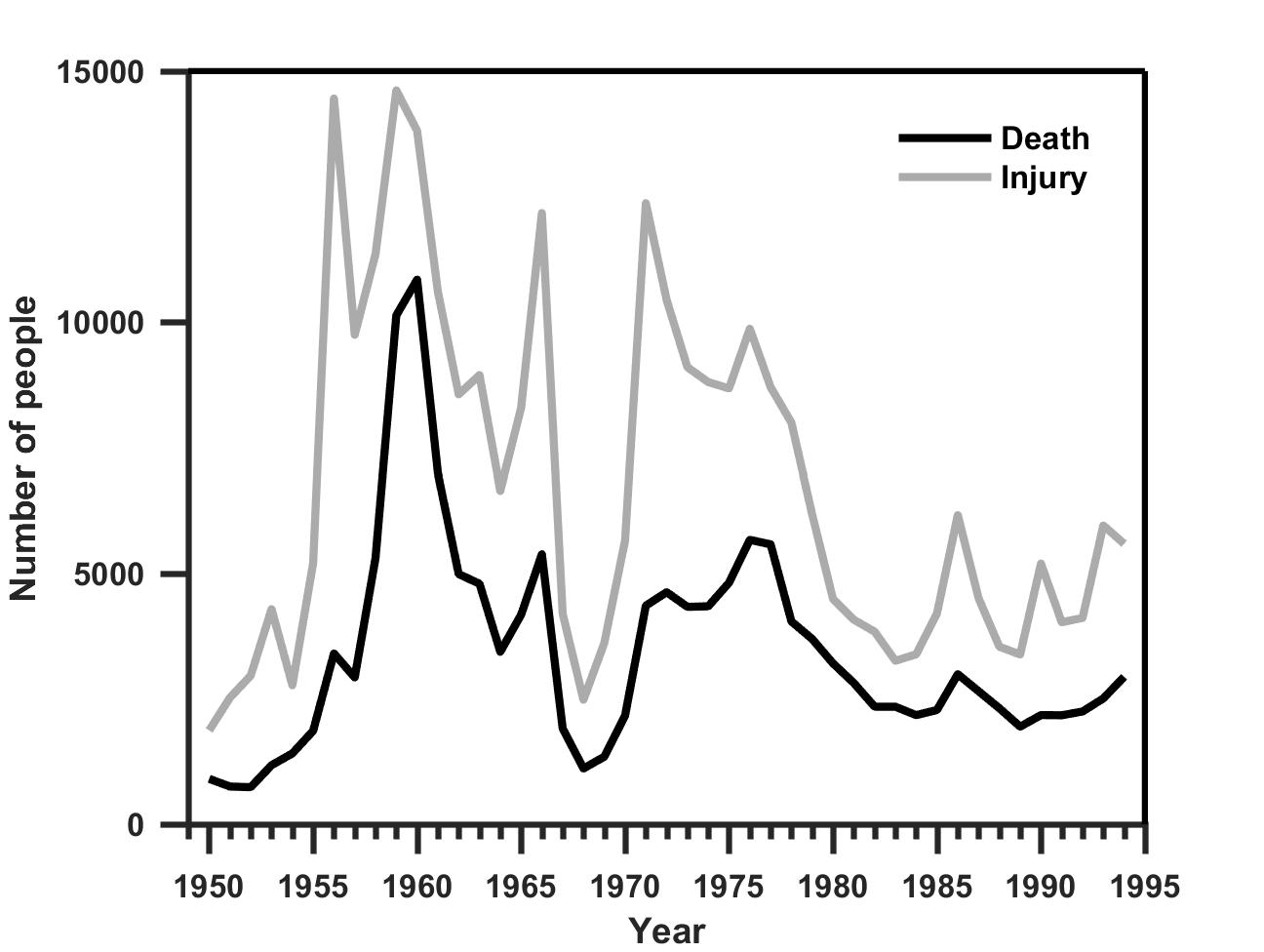


**Figure S1.** Twentieth century trends in fire-related casualties in China. Number of casualties and serious injuries per year (1950–1994) caused by fire in China.


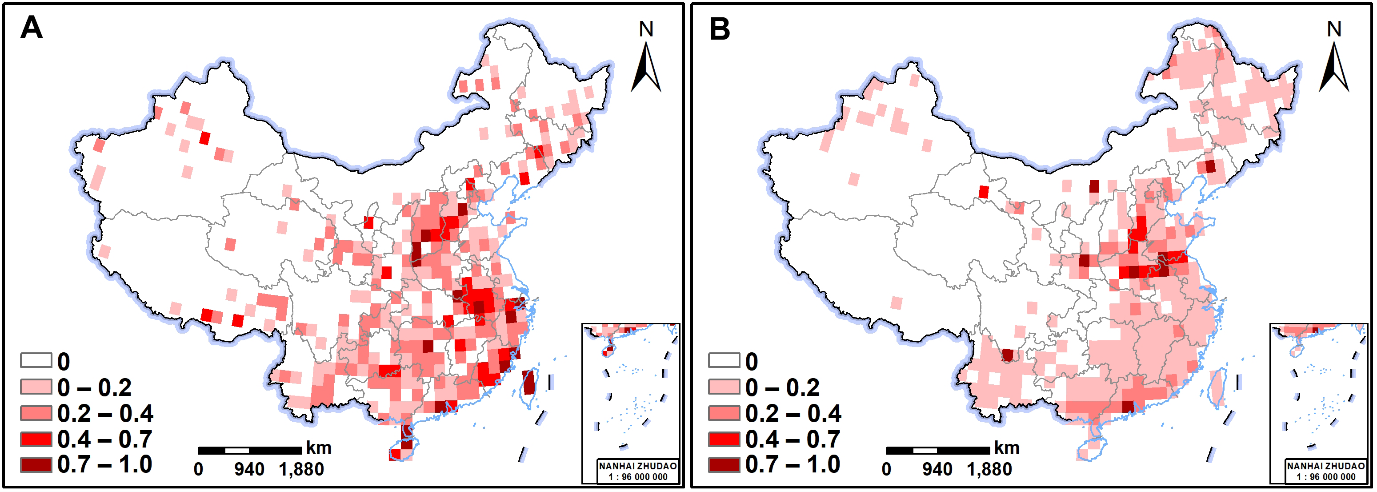


**Figure S2.** The spatial distribution of normalized fire frequency (0 means no fire and 1 means the highest fire frequency) of CFHA data in 1990–1994 (a) and MODIS data in 2001–2005 (b). The correlation coefficient between (a) and (b) is 0.37 (*p* < 0.001).


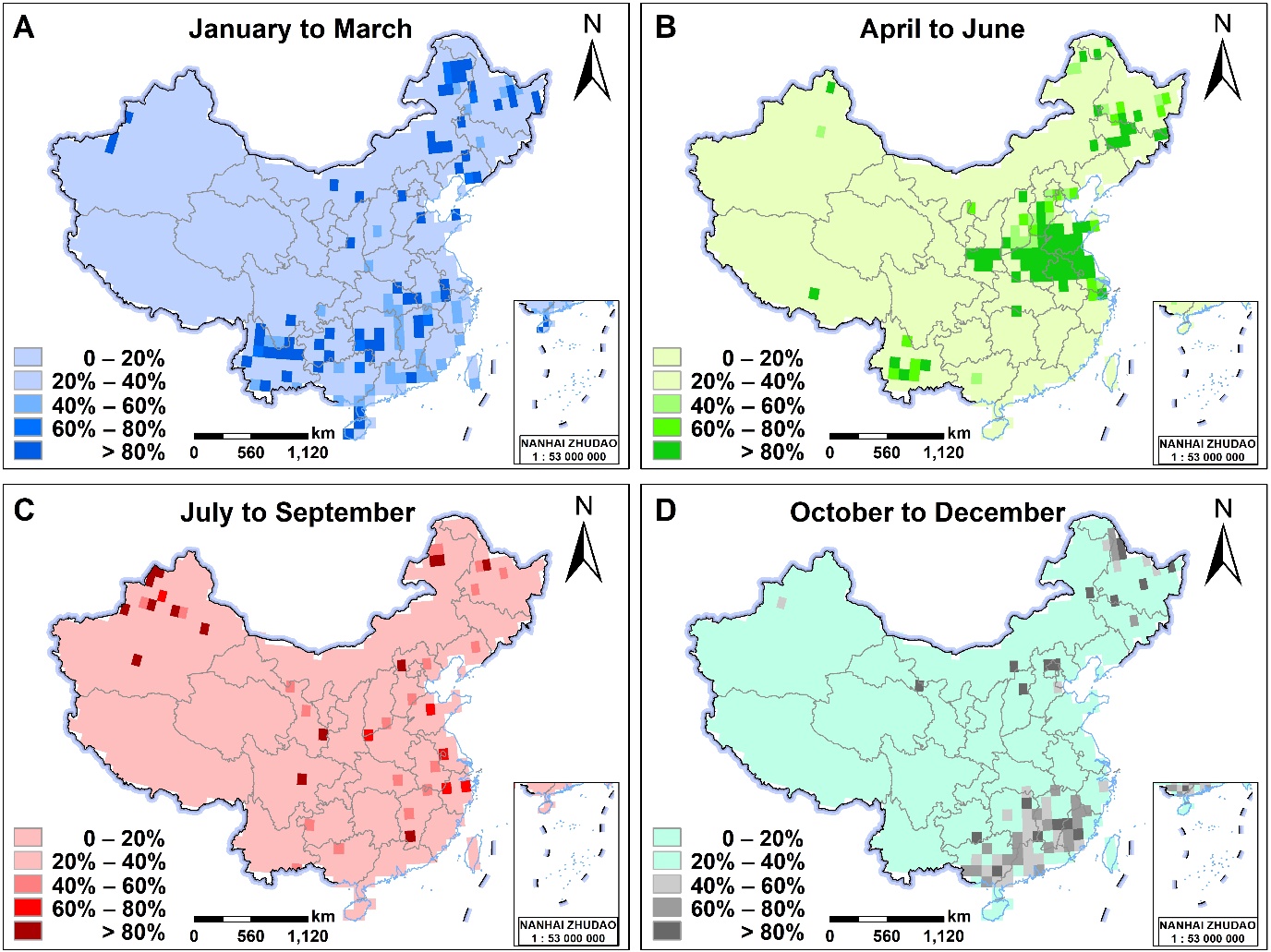


**Figure S3.** Fire seasonality map of China (2001–2005) derived from MODIS fire product. It is generally consistent with the fire seasonality map from CFHA (see Fig. 2 in the main text).


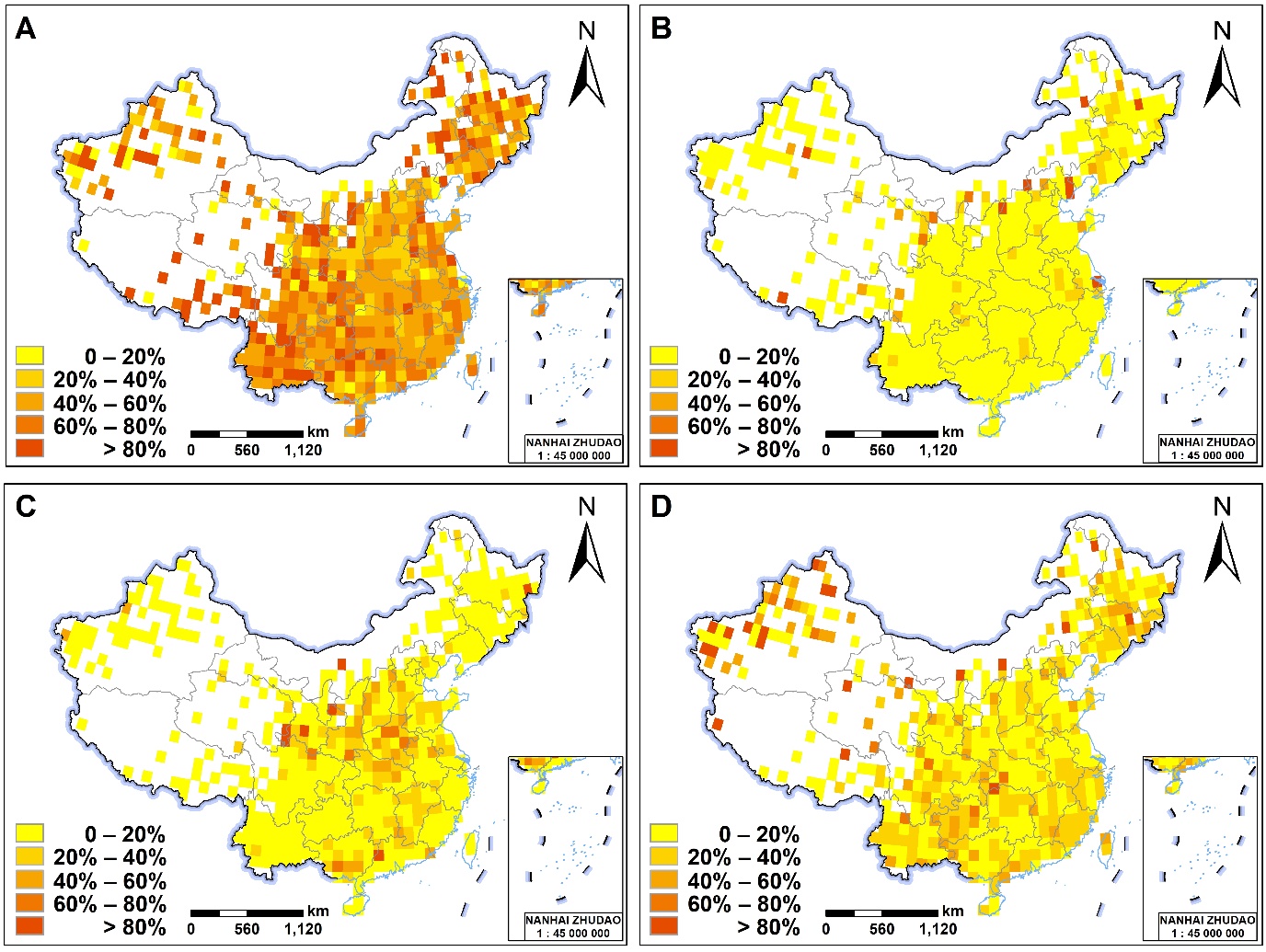


**Figure S4.** Cause of fires in China. Percentage of fires (1901–1994) per grid point in the CFHA for each fire cause category. (A) Anthropogenic fire, (B) natural fire, (C) war fire, and (D) others.


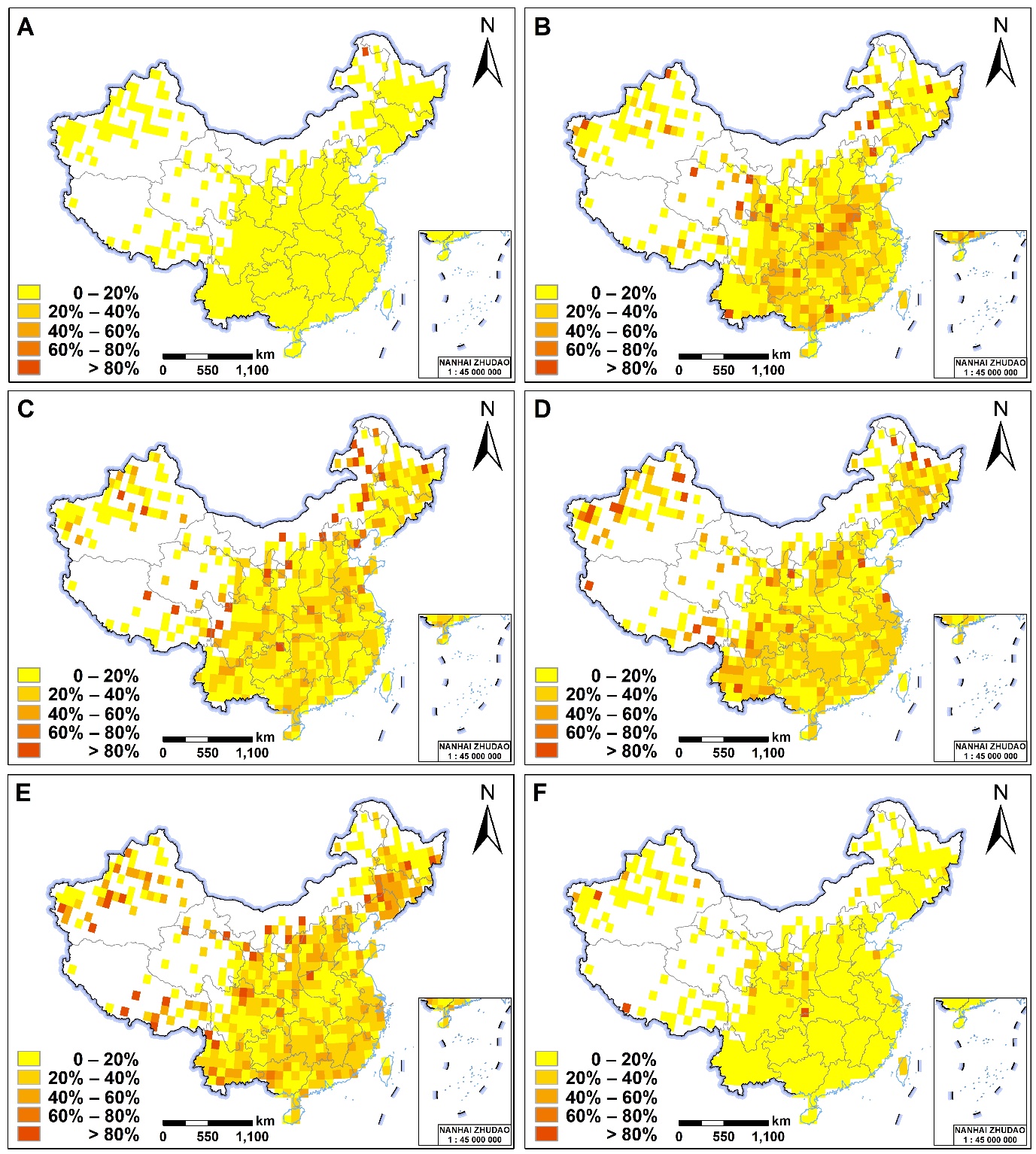


**Figure S5.** Fire severity maps of China. Percentage of fires (1901–1994) per grid point in the CFHA for each fire severity level. (A) Super fire, (B) Very large fire, (C) Large fire, (D) Medium fire, (E) Small fire, and (F) Unknown.

**Figure S6.** Twentieth century fire chronologies in China. Annual number of fires (1901–1994) in China caused by anthropogenic sources (A), natural sources (B), wars (C), and other causes (D). Total fire number in China (1901–1994) with contribution of individual severity levels (E).

**Figure S7.** Twentieth century fire chronologies in large and small cities in China. Annual number of fires (1901–1994) in China for large and small cities.


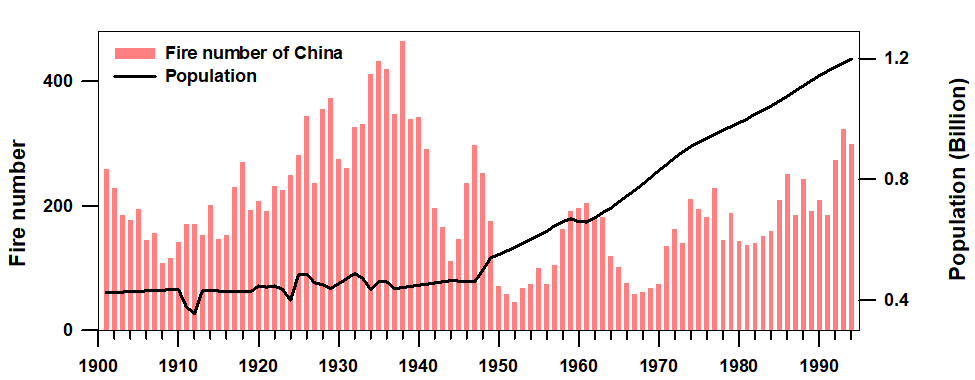


**Figure S8.** Fire number and population in China (1901–1994).

**Figure S9.** Seasonal climate variability for four subregions of China. Seasonal temperature (bars) and precipitation (line) variability (1901–1994) for four subregions of China. Subregions are indicated by red boxes in Fig. 2. (Southwest (96°–107°E, 21°–27°N), Northeast (115°–133°E, 40°–53°N), Central (103°–110°E, 27°–33°N) and South (107°–117°E, 21°–26°N)). Temperature and precipitation data were derived from gridded 1° CRU TS 3.24 temperature fields and averaged over the corresponding subregions. Seasons include winter (January–March), spring (April–June), summer (July–September), and autumn (October–December).


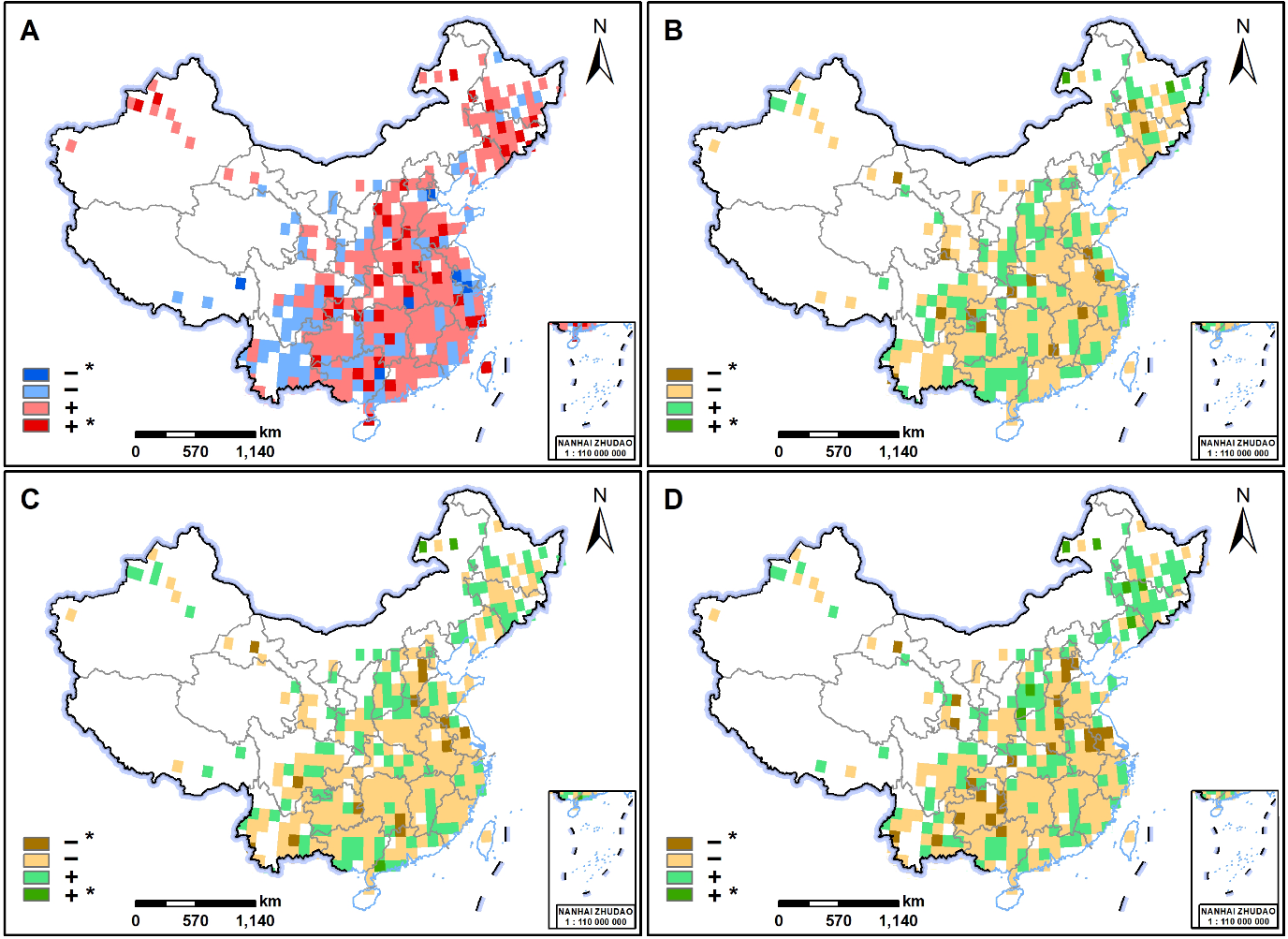


**Figure S10.** Climate anomalies during fire years from 1901 to 1994 across China (1° × 1°) using the superposed epoch analysis method. Annual temperature (A), SPEI (B), precipitation (C), and PDSI (D) anomalies during the 20 largest fire years in the CFHA (1901–1994; 1° × 1°). Blue/yellow colors indicate negative (cold or dry) anomalies, red/green colors indicate positive (warm or wet) anomalies. Dark colors indicate statistical significance at the 95% level as identified based on a two-sample *t*-test.


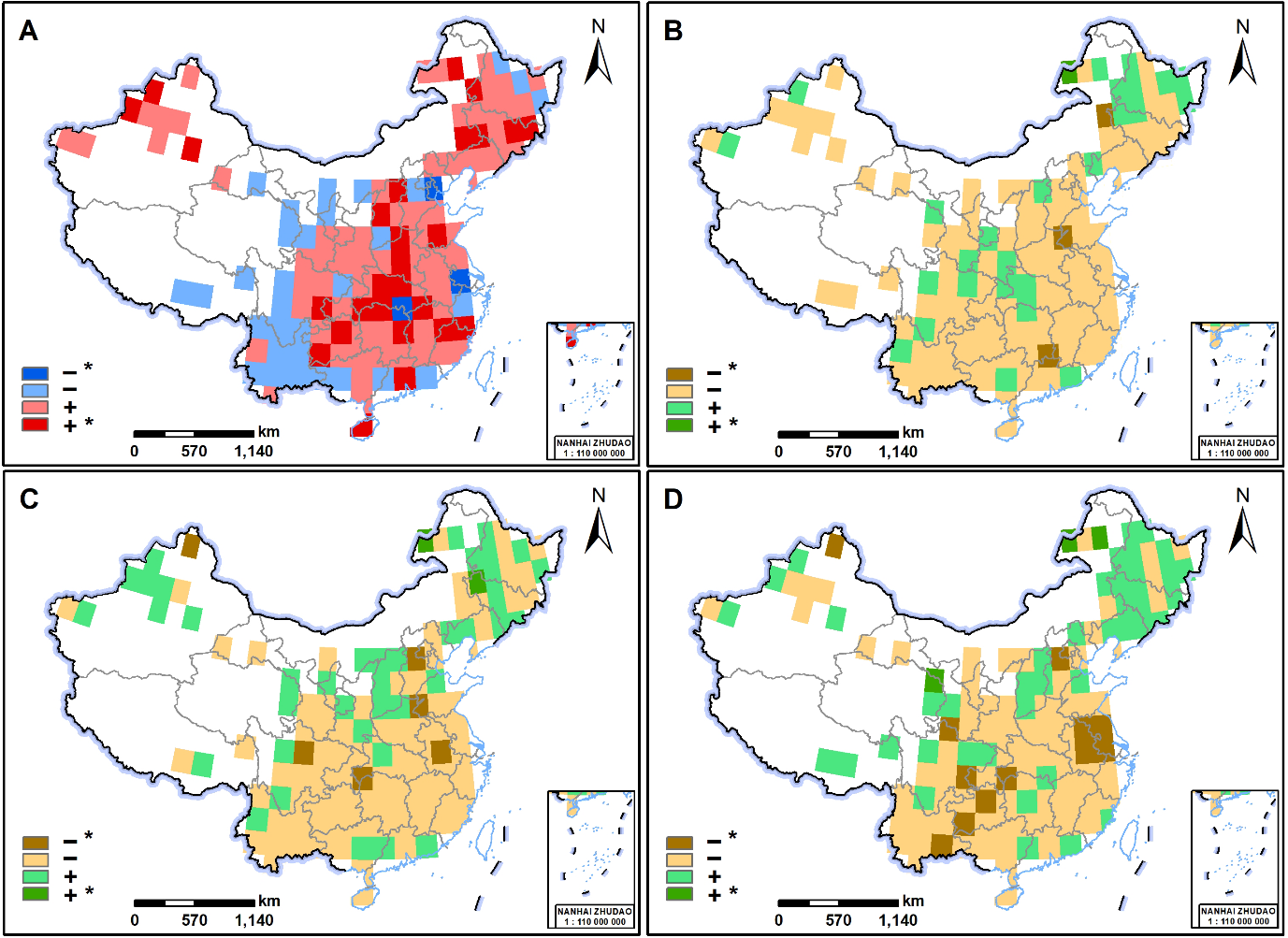


**Figure S11.** Climate anomalies during fire years from 1901 to 1994 across China (2° × 2°) using the superposed epoch analysis method. Annual temperature (A), SPEI (B), precipitation (C), and PDSI (D) anomalies during the 20 largest fire years in the CFHA (1901–1994; 2° × 2°). Blue/yellow colors indicate negative (cold or dry) anomalies, red/green colors indicate positive (warm or wet) anomalies. Dark colors indicate statistical significance at the 95% level as identified based on a two-sample *t*-test.

**Figure S12.** Z-scores of China fire chronology (CFC), mean annual temperature, and annual precipitation days during 1901–1994, and z-scores of fire-related annual economic loss during 1950–1994.

**Figure S13.** Annual and seasonal climate anomalies during fire years from 1901 to 1994 in Northeast China using the superposed epoch analysis method. Annual and seasonal temperature (A) and SPEI (B) anomalies determined by a composite analysis of the 20 largest fire years in Northeast China during 1901–1994, indicated by red boxes in Fig. 2.


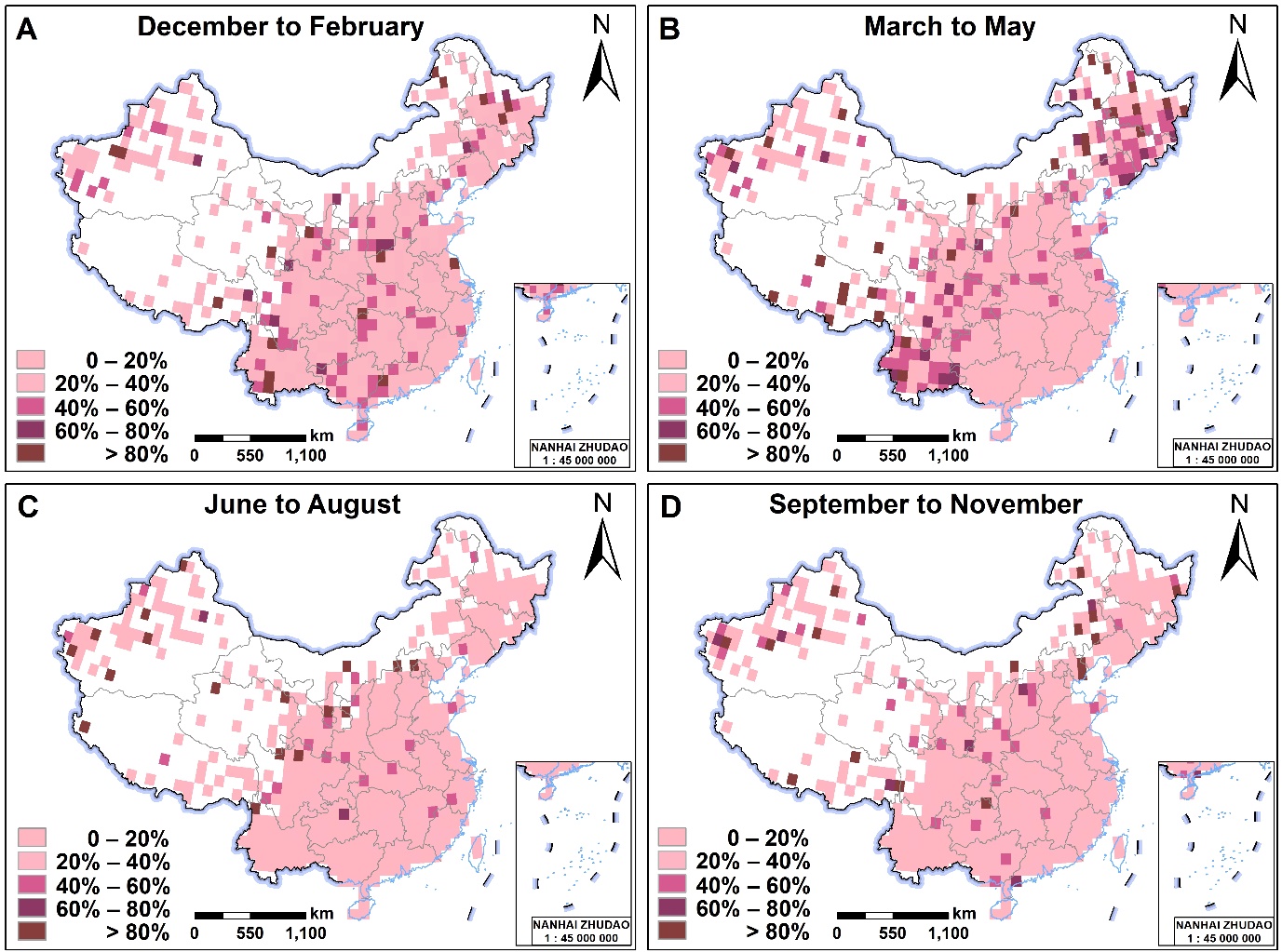


**Figure S14.** Distribution of fire percentages during the period of 1901–1994 in the China Fire Historical Atlas (CFHA) following the monsoon-based seasonal definition of winter (previous December to February), pre-monsoon (March to May), monsoon (June to August), post-monsoon (September to November) periods.


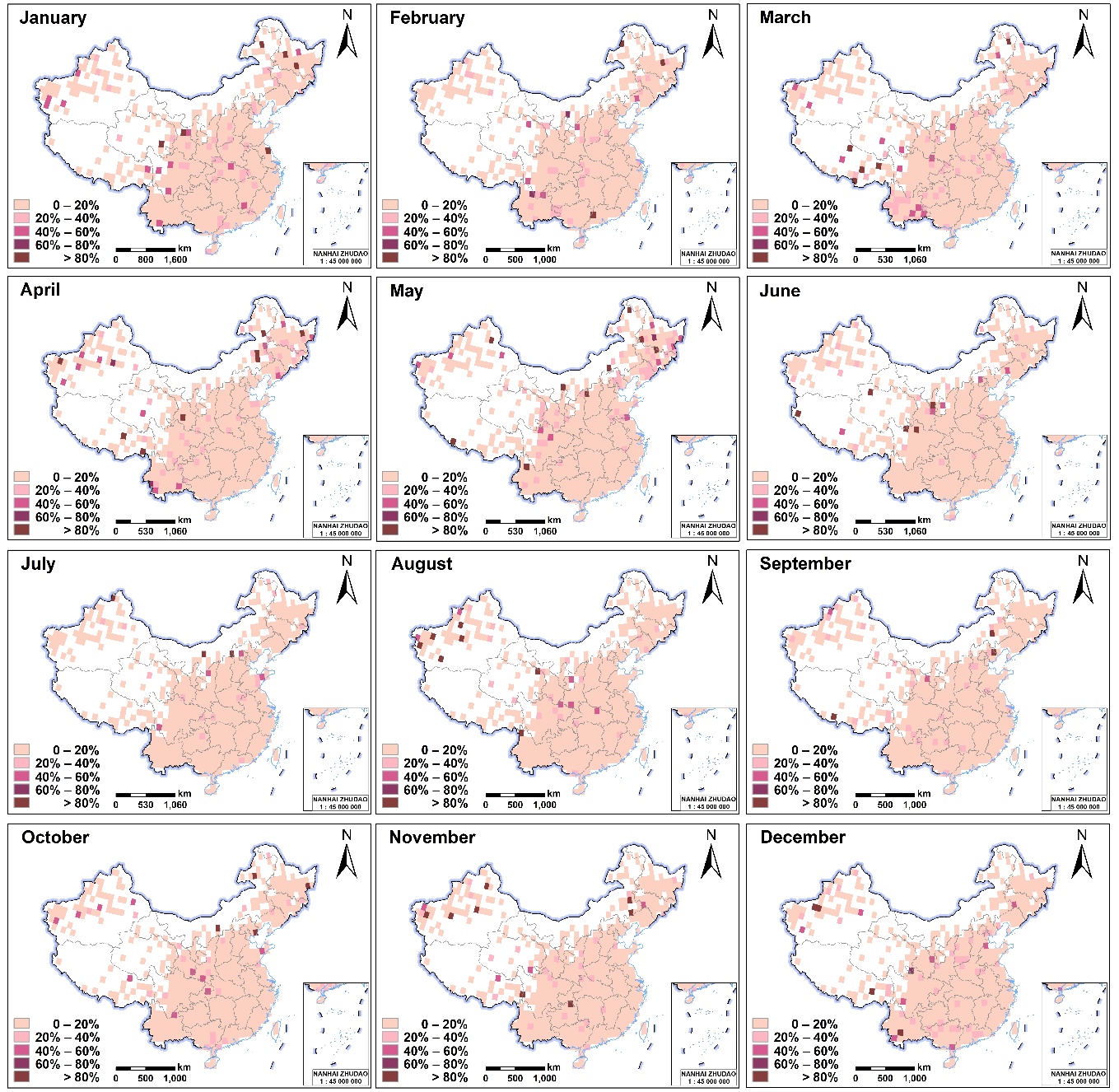


**Figure S15.** Monthly distribution of the percentages of urban fires during the period of 1901–1994.


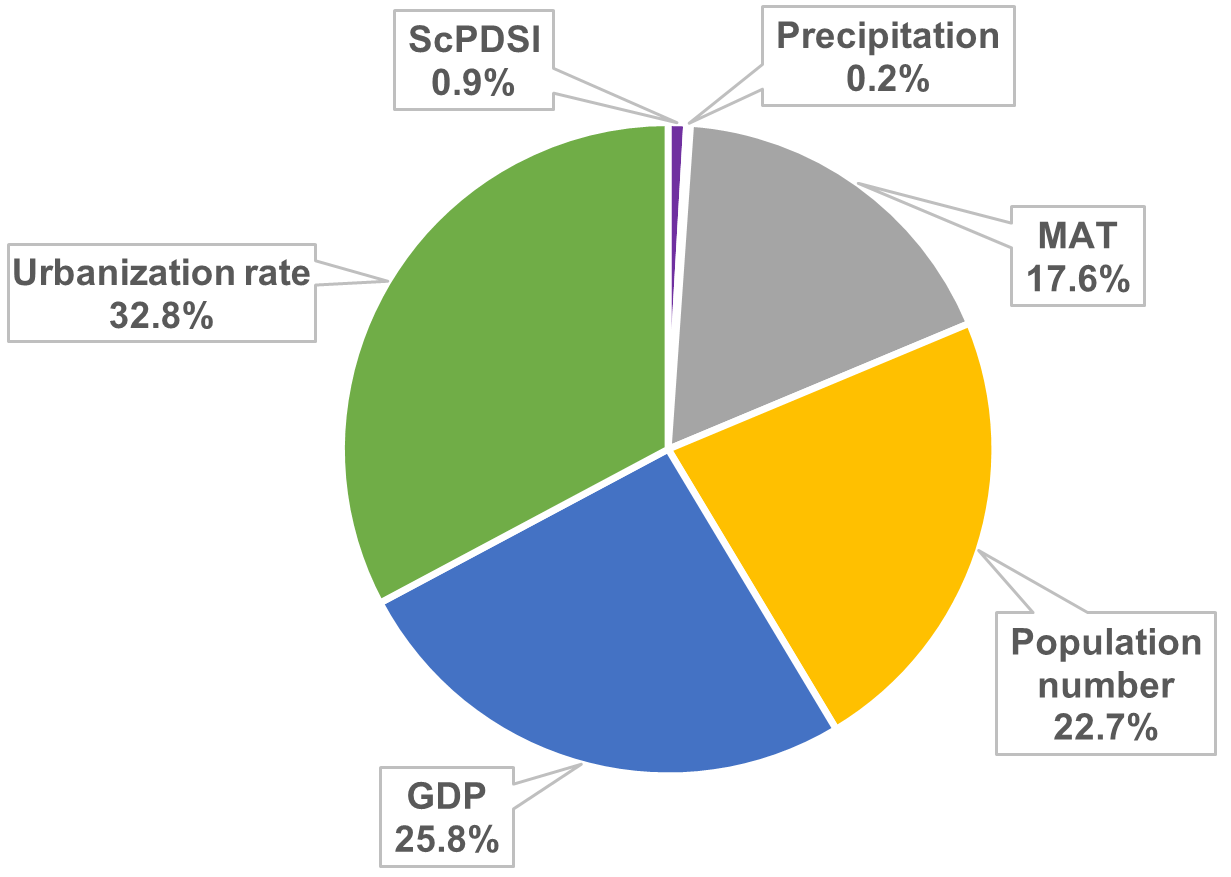


**Figure S16.** Relative importance of urbanization, economic growth, population growth and climate factors (MAT, precipitation and scPDSI) on fire activity from 1950 to 1994.


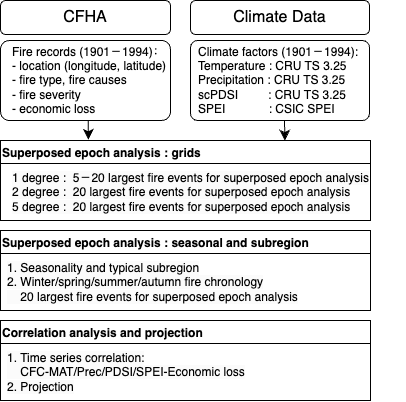


**Figure S17.** Framework diagram of data analysis.

**Table S1.** Pearson correlations of the annual CFC and fire number with the maximum, minimum and mean temperature.

|  |  | Maximum  Temperature | Minimum  Temperature | Mean  Temperature |
| --- | --- | --- | --- | --- |
|  |  | January–December | January–December | January–December |
| 1901–1994  (Annual) | CFC | **0.43***** | **0.66***** | **0.58***** |
|  | Fire Number | **0.19*** | **0.28**** | **0.23**** |
| 1950–1994  (Annual) | CFC | **0.31**** | **0.65***** | **0.56***** |
|  | Fire Number | **0.32**** | **0.65***** | **0.57***** |

**p* < 0.1, ***p* < 0.05, ****p* < 0.001

**Table S2.** Pearson correlation coefficients between the first-order differenced mean annual temperature and number of fires in six large cities with long continuous records and continuous fire records.

| City (start of meteorological record) | start–1949 | 1950–1994 | start–1994 |
| --- | --- | --- | --- |
| **Beijing (1901)** | 0.09 | **0.36**** | 0.13 |
| **Shanghai (1901)** | 0.00 | **0.39***** | 0.04 |
| **Guangzhou (1912)** | **0.31*** | **0.57***** | **0.33***** |
| **Taiyuan (1916)** | 0.00 | **0.57***** | **0.51***** |
| **Xiamen (1915)** | –0.23 | **0.24** | **0.19*** |
| **Wenzhou (1924)** | 0.12 | **0.49***** | **0.38***** |

**p* < 0.1, ***p* < 0.05, ****p* < 0.01

**Table S3.** Fire, temperature and fire-related economic loss interaction. Pearson correlation coefficients among economic loss, fire number, and mean annual temperature from 1950 to 1994.

|  | Fire number | Mean annual temperature |
| --- | --- | --- |
| Economic loss | **0.77***** | **0.57***** |
| Fire number |  | **0.57***** |

****p* < 0.001

**Table S4.** Projection models. Coupled Model Intercomparison Project Phase 6 (CMIP6) outputs of 46 modeling groups.

|  | CMIP6 model name | Used member | Horizontal resolution (longitude × latitude) |
| --- | --- | --- | --- |
| 1 | ACCESS-CM2 | 3 | 0.8° × 0.533° |
| 2 | ACCESS-ESM1-5 | 3 | 0.806° × 0.533° |
| 3 | AWI-CM-1-1-MR | 1 | 1.067° × 1.067° |
| 4 | BCC-CSM2-MR | 1 | 0.889° × 0.889° |
| 5 | CAMS-CSM1-0 | 2 | 0.889° × 0.889° |
| 6 | CESM2 | 5 | 1.067° × 0.8° |
| 7 | CESM2-WACCM | 3 | 1.067° × 0.8° |
| 8 | CIESM | 1 | 1.067° × 0.8° |
| 9 | CMCC-CM2-SR5 | 1 | 1.067° × 0.8° |
| 10 | CNRM-CM6-1 | 6 | 0.711° × 0.711° |
| 11 | CNRM-CM6-1-HR | 1 | 2° × 2° |
| 12 | CNRM-CM6-1-HR-f2 | 1 | 2° × 2° |
| 13 | CNRM-CM6-1-f2 | 6 | 0.711° × 0.711° |
| 14 | CNRM-ESM2-1 | 5 | 0.711° × 0.711° |
| 15 | CNRM-ESM2-1-f2 | 5 | 0.711° × 0.711° |
| 16 | CanESM5 | 50 | 0.356° × 0.356° |
| 17 | CanESM5-CanOE | 3 | 0.356° × 0.356° |
| 18 | CanESM5-CanOE-p2 | 3 | 0.356° × 0.356° |
| 19 | CanESM5-p1 | 25 | 0.356° × 0.356° |
| 20 | CanESM5-p2 | 25 | 0.356° × 0.356° |
| 21 | EC-Earth3 | 7 | 1.422° × 1.422° |
| 22 | EC-Earth3-Veg | 5 | 1.422° × 1.422° |
| 23 | FGOALS-f3-L | 1 | 1° × 0.8° |
| 24 | FGOALS-g3 | 4 | 0.444° × 0.5° |
| 25 | FIO-ESM-2-0 | 3 | 1.067° × 0.8° |
| 26 | GFDL-ESM4 | 1 | 1° × 0.8° |
| 27 | GISS-E2-1-G | 1 | 0.5° × 0.4° |
| 28 | GISS-E2-1-G-p3 | 1 | 0.5° × 0.4° |
| 29 | HadGEM3-GC31-LL | 4 | 0.8° × 0.533° |
| 30 | HadGEM3-GC31-LL-f3 | 4 | 0.8° × 0.533° |
| 31 | INM-CM4-8 | 1 | 0.667° × 0.5° |
| 32 | INM-CM5-0 | 1 | 0.667° × 0.5° |
| 33 | IPSL-CM6A-LR | 6 | 0.794° × 0.4° |
| 34 | KACE-1-0-G | 3 | 0.8° × 0.533° |
| 35 | MCM-UA-1-0 | 1 | 0.444° × 0.267° |
| 36 | MIROC-ES2L | 1 | 0.356° × 0.356° |
| 37 | MIROC-ES2L-f2 | 1 | 0.356° × 0.356° |
| 38 | MIROC6 | 50 | 0.711° × 0.711° |
| 39 | MPI-ESM1-2-HR | 2 | 1.067° × 1.067° |
| 40 | MPI-ESM1-2-LR | 10 | 0.533° × 0.533° |
| 41 | MRI-ESM2-0 | 2 | 0.889° × 0.889° |
| 42 | NESM3 | 2 | 0.533° × 0.533° |
| 43 | NorESM2-LM | 1 | 0.533° × 0.4° |
| 44 | NorESM2-MM | 1 | 1.067° × 0.8° |
| 45 | UKESM1-0-LL | 5 | 0.8° × 0.533° |
| 46 | UKESM1-0-LL-f2 | 5 | 0.8° × 0.533° |


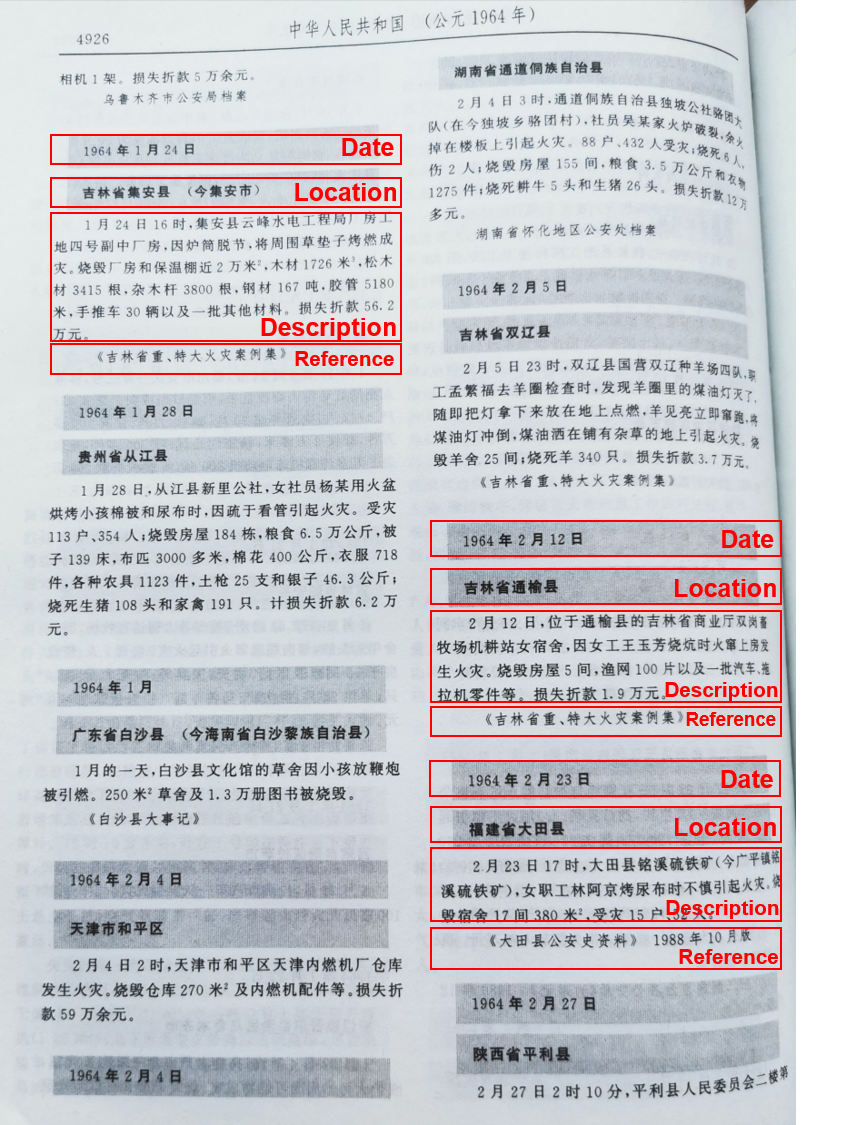


**Photo S1.** Examples about the fire descriptions in documentary records.

**Movie S1.**

The China Fire History Atlas.
